# Clinical Trial Data Transparency in the EU: Is the New Clinical Trials Regulation a Game-Changer?

**DOI:** 10.1007/s40319-023-01329-4

**Published:** 2023-05-04

**Authors:** Żaneta Zemła-Pacud, Gabriela Lenarczyk

**Affiliations:** 1 Dr.; Department of Polish and European Industrial Property Law, Polish Academy of Sciences, Warsaw, Poland; 2 Dr.; Department of Private Law, Institute of Law Studies, Polish Academy of Sciences, Warsaw, Poland

**Keywords:** Clinical trial data, CTIS, Clinical Trials Regulation, Commercially confidential information, Clinical trial data transparency

## Abstract

The benefits of access to clinical trial data are related to their inestimable value from the perspective of clinical trial participants, society as a whole, public health systems and scientific progress. In light of the development of innovative data analysis technologies, access to raw clinical trial data opens up an ever-widening array of possibilities: it can profoundly facilitate machine data analysis for, *inter alia*, hypothesis generation, risk modelling, counterfactual simulation and – finally – drug repurposing and development. The enactment of the new Clinical Trials Regulation (EU) No. 536/2014 (CTR) and introduction of the Clinical Trials Information System (CTIS) were heralded as ensuring a level of transparency in clinical trials that is sufficient to contribute to protecting public health and fostering the innovation capacity of European medical research, while recognizing the legitimate economic interests of sponsors. This paper presents the hitherto binding rules for the disclosure of clinical trial data and, against this background, their new framework, introduced by the CTR. In addition to assessing whether the CTR’s objectives are fulfilled, this paper examines whether the latest changes impact the hitherto existing rules on protection of regulatory data via regulatory exclusivities. Finally, it points out concerns regarding whether data gathered in the CTIS can be efficiently used by innovative data analysis technologies for further processing for both commercial and non-commercial purposes.

## Introduction

Clinical trial data constitute a key part of the marketing authorization dossier of a medicinal product submitted for assessment of its safety and efficacy. The benefits of access to these data have long been recognized by the global community. Governments,^1^ international organizations^2^ and researchers^3^ specifically point to the value of clinical trial data from the perspective of clinical trial participants, society as a whole, public health systems and scientific progress. First and foremost, such access is essential for an independent scientific verification of a product’s safety and efficacy; it further has a strong ethical rationale as it can reduce the duplication of clinical trials (especially those that have been unsuccessful); it allows assessment of the transparency of a medicinal product’s authorization process; and, finally, supports the building of general trust towards clinical trials and medicinal products among patients. In light of the development of innovative data analysis technologies, access to raw clinical trial data opens up a wide array of possibilities: it can profoundly facilitate machine data analysis for, *inter alia*, hypothesis generation, risk modelling, counterfactual simulation, and drug repurposing and development.^4^

The new Clinical Trials Regulation (EU) No. 536/2014^5^ (CTR) became applicable in January 2022, with one of its key goals being increased transparency in the area of clinical trials.^6^ The enactment of the CTR and the introduction of the Clinical Trials Information System (CTIS), central to fulfilling this goal, were heralded as ensuring a level of transparency in clinical trials that is sufficient to contribute to protecting public health and fostering the innovation capacity of European medical research, while recognizing the legitimate economic interests of sponsors.^7^ Concurrently, promises of unparalleled transparency levels raised concerns on the part of research-based pharmaceutical companies, which are uncertain of the depth of changes to existing rules on protection for their confidential clinical trial data and a potentially negative impact of such changes on their competitive position.^8^

Moreover, at first glance, the noticeable, gradually ongoing change towards greater access to such data within the European Union – first reflected in the European Medicines Agency (EMA)’s policies regarding transparency and access to clinical trial data^9^ and now more firmly established by the wording of Regulation No. 536/2014 – seemed to go hand-in-hand with the rise of innovative data analysis technologies. The ever-expanding data not only benefit from, but in fact begin to require Artificial Intelligence (AI)-powered capabilities to gather, normalize and analyze them in the interests of both public healthcare and drug R&D.^10^ However, the limited amounts of data available for model training constitute considerable restraints to efficient implementation of such AI-driven projects.

This paper presents the hitherto binding rules for the disclosure of clinical trial data and, against this background, their new framework, introduced by the CTR. By carrying out a detailed analysis of the new rules for publishing clinical trial data in the CTIS, particularly their scope, timing of publication, and relevance for various stakeholders in the pharmaceutical sector, this paper aims to assess whether they fulfil the CTR’s objectives. It also examines whether these latest changes impact the existing rules on protection for regulatory data via regulatory exclusivities. Finally, concerns are pointed out regarding whether data gathered in the CTIS can be efficiently used by innovative data analysis technologies for further processing for both commercial and non-commercial purposes. As the new rules on clinical trial data disclosure only came into force at the end of January 2022, and thus many questions remain unanswered, this paper maps out areas for further research. As it specifically focuses on rules for the disclosure of clinical trial data, this paper does not analyze other protective regimes that can be relevant for the data: in particular, the protection of undisclosed know-how via trade secrecy and the protection of individuals’ privacy in accordance with Regulation (EU) 2018/1725.^11^

## Importance of Clinical Trials Results and the EU Policy in this Area

Clinical trials are an essential part of research and development of new medicines, both based on new active substances and reformulations of known medicines. In the latter case, the development process continues after a medicine’s approval, with further studies conducted to examine long-term outcomes, further indications, and safety and efficacy in routine usage. Importantly, information collected in the course of clinical trials can benefit patients, healthcare professionals, researchers and the public, regardless of a clinical trial’s outcome. It can improve decision-making related to recruitment for a trial, improve collaboration across the research community, avoid duplication of unsafe or unsuccessful trials, drive efficiencies in trial design and conduct, and provide greater insight for trial participants and the public to support healthcare decisions while enhancing trust between patients, regulators and sponsors.^12^ Some consider data-sharing to be almost a moral imperative to research subjects.^13^ Others argue that access to drug safety and efficacy data is not a matter of weighing the interest of patients or consumers in accessing data and the business interest in secrecy, but rather that these data constitute public goods.^14^ In any case, the legitimate interests of sponsors must be recognized, too, as clinical trials constitute the most time-consuming and costly part of drug research and development.^15^

Before a clinical trial can be conducted in the EU, it must be approved by the national competent authorities and ethics committees in each Member State (each site) where a trial is planned to be carried out. This guiding principle of conducting clinical trials within the EU has applied since the entry into force of the Clinical Trials Directive (CTD)^16^ and remains valid under the new Regulation.^17^ Under the Directive, all other activities required of the sponsor – such as the registration and posting of trial results – were also separate processes, carried out separately in each Member State. The Directive remained applicable until 31 January 2023, from which date all clinical trial applications have to be submitted via the CTIS, in line with the requirements of the Clinical Trials Regulation.^18^ Changes introduced by the CTR do not affect the Member State’s authority to issue regulatory approval to run a clinical trial. Instead, by establishing the Clinical Trials Information System, the Regulation streamlines the application and supervision of clinical trials as well as their public registration. All clinical trial sponsors will use the same system and follow the same process for authorization. Whereas the Member States remain solely responsible for the authorization and oversight of clinical trials, the European Medicines Agency is responsible for maintaining the CTIS. The European Commission’s role is to oversee the implementation of the CTR.

When discussing access to clinical trial data, one must differentiate two stages at which disclosure could be granted: the pre-registration phase (including the duration of a clinical trial) and the post-registration phase, once a dossier comprising data on the safety and efficacy of a medicinal product is submitted to regulatory authorities and a decision on its authorization is reached. Within the first stage, the issue of availability of data relates mainly to trial registration, as well as reporting and publication of trial results upon completion of their respective phases, whereas access within the second stage concerns clinical study reports (CSRs) and individual patient data (IPD).^19^ The CTR introduces strict transparency requirements, discussed in detail below, throughout both of these stages.

In the EU, transparency requirements of regulatory data submitted for authorizing medicines via a centralized route have generally been established in multiple documents at many levels: in the Charter of Fundamental Rights of the European Union,^20^ the Treaty on the Functioning of the European Union (TFEU),^21^ Regulation (EC) No. 1049/2001 regarding public access to European Parliament, Council and Commission documents,^22^ Regulation (EC) No. 1901/2006 on medicinal products for paediatric use,^23^ Regulation (EC) No. 726/2004 laying down Community procedures for the authorization and supervision of medicinal products for human and veterinary use and establishing a European Medicines Agency,^24^ and Directive 2001/20/EC on the approximation of the laws, regulations and administrative provisions of the Member States relating to the implementation of good clinical practice in the conduct of clinical trials on medicinal products for human use. There are also policies and guidelines issued by the European Medicines Agency (EMA): specifically, Policy 0043 for request-based access to documents related to medicinal products^25^ and Policy 0070 for publication of clinical trial data,^26^ as well as the guidance document on the identification of commercially confidential information (CCI) and personal data within the structure of the marketing authorization application, issued by the Heads of Medical Agencies (HMA) together with the EMA.^27^ Whereas the legal and ethical principles of efficiency, accountability, and transparency in clinical research are generally accepted in the majority of countries all around the globe,^28^ before the CTR came into force specific requirements in this area varied by country. Thus, access to the dossiers of medicines approved in the decentralized procedure could be described as arbitrary and depended on the individual approach of the Member States’ authorities in possession of the requested documents.^29^ The guidance document on CCI, adopted in 2012, aimed at reducing the inconsistencies in the National Competent Authorities’ (NCAs) decisions as to what should be considered to be commercially confidential under all procedures (the national, mutual recognition, decentralized and centralized procedures); however, not all the NCAs applied the established guidelines.^30^

The right of access to documents of the institutions, bodies, offices and agencies of the European Union is enshrined in Art. 42 of the Charter of Fundamental Rights of the European Union and guaranteed by the Treaty on the Functioning of the European Union (Art. 15). Within this framework, access to clinical trial data contained in documents held by the EMA is fundamentally based on Regulation No. 1049/2001, which gives effect to the abovementioned primary EU law. Such access is, however, subject to exceptions stipulated in Art. 4 of Regulation 1049/2001. In accordance with Art. 4(2), institutions are to refuse access to a document where disclosure would undermine, *inter alia*, the protection of the commercial interests of a natural or legal person, including intellectual property, unless there is an overriding public interest in disclosure of the document in question. Under a policy governing access to its documents, applied until 2010, the EMA generally refused^31^ access to documents contained in the dossier submitted by a company for the purposes of an MA, as well as to unpublished trial reports and their corresponding protocols.^32^

In 2010, the confidentiality paradigm began to shift towards transparency when the EMA published its Policy 0043 which allowed anyone to request clinical and other documents from the EMA, thereby authorizing the Agency to release data reactively. The new transparency framework was completed five years later with the EMA’s Policy 0070. The latter introduced an obligation to publish regulatory clinical documents after a medicinal product has received marketing authorization and conferred the EMA the authority to proactively publish data on an online sharing portal. While Policy 0043 guaranteed the right to access dossiers of already centrally authorized drugs, Policy 0070 ensured the release of clinical trial data of newly centrally authorized drugs. The EMA’s policies were drafted with the objective of allowing medicine developers, the scientific community and other third parties access to detailed clinical trial data in order to learn from past successes and failures, develop new knowledge in the interest of public health, verify the original analyses and conclusions, and to conduct further analysis. It allowed academic and non-commercial research users to download clinical reports in searchable formats, as well as anonymized individual patient data, which are defined as “the individual data separately recorded for each participant in a clinical study”.^33^

Importantly, before Policy 0070, there was no possibility of automatic or facilitated access to clinical trial data. Any clinical trial data that were of interest to third parties needed to be requested in a separate application, submitted either to an NCA or to the EMA.^34^

Thereby, the Agency adopted an open access approach to clinical trial data *on which its regulatory decisions are based*, subject to the exception of protecting commercially confidential information. Applicants who submitted dossiers in the marketing authorization (MA) procedure were able to redact CCI from their documents if the EMA found their justification of the redactions sufficient and adequate.^35^ Nevertheless, the status of Clinical Study Reports (CSRs), submitted as part of the MA application, remained the axis of dispute between the EMA and pharmaceutical companies.^36^ A CSR is a report on an individual study of an investigational medicinal product, in which the clinical and statistical description, presentations, and analyses are integrated.^37^ The importance of CSRs stems from the vast amounts of information they contain, e.g. on the conduct, adverse events and outcome of a clinical trial. Concurrently, CSRs are referred to in the literature as a “hitherto mostly hidden and untapped source of detailed and exhaustive data on each trial”.^38^ The open access approach to CSRs was said to create two main risks in terms of data-sharing: first, to participants’ privacy and, second, to individual companies with regard to their commercial interests. The latter risk was mainly emphasized by the sponsors of clinical trials, who feared that copied CSRs might, in theory, be used for the purposes of receiving regulatory approval in jurisdictions with limited regulatory data protection laws.^39^ At that time, there seemed to be *some* empirical basis to support this concern, as research conducted in 2013 has shown that the majority of early requests to the EMA to access clinical trials data had been made by pharmaceutical companies, lawyers and consultants – not academic investigators.^40^ Additionally, a survey conducted by the Agency among users of a clinical trial data website eight months after its launch found that groups related to the pharmaceutical industry used the data not only to check compliance with the Agency’s requirements for CSRs, but also as a benchmark against other companies and to be aware of competitors’ activities.^41^ Ultimately, the pharmaceutical companies failed to prove to the CJEU that such a risk is high enough to warrant a refusal of access to CSRs.^42^

The publication process of clinical reports introduced by Policy 0070 was based on the terms of use governing the access to and use of clinical reports. By accepting the terms of use, users of the clinical trial data website^43^ agreed to only use the clinical reports provided in the EMA’s database for academic and non-commercial research purposes. Further, the clinical reports may not be used to support an MA application/extensions or variations to an MA for a product anywhere in the world, nor to make any unfair commercial use of the clinical reports.^44^ There are two visible limitations to the effective use of this policy: first, there is a question of enforcement and sanctions following non-compliance. Even though the sponsor may, in theory, seek to use the terms of use as evidence of a wrongful acquisition of data, it would need to obtain information about the identity of the user, which the EMA has historically declined to provide.^45^ Second, the contractual provision only binds the initial user. If the data become public, the enforcement issue becomes yet more complex. These limitations were to be mitigated by placing watermarks on published clinical report data.

The legal status of CSRs was inconsistent across Europe: under the old Clinical Trials Directive, CSRs were neither determined to be confidential, nor subject to publication. Concurrently, the EMA in its Policies 0043 and 0070 clearly guaranteed the right to access to CSRs, which was confirmed by the CJEU judgments stating that CSRs do not constitute CCI in their entirety.^46^

A study conducted during 2018 and 2019 revealed that, in practice, access to CSRs was only granted in some European countries.^47^ Whereas CSRs were not considered commercially confidential information by the CJEU (see cases mentioned above), nor by the HMA and EMA,^48^ some NCAs applied a different degree of transparency for drugs approved through national approval routes. In particular, German, Finnish and Polish regulators refused to grant access to the reports, pointing to the confidentiality of data.^49^ Thus, it can be observed that the discrepancy between a high level of transparency applicable to centrally approved drugs and various degrees of transparency applicable to drugs authorized via national routes led to unequal standards of access to clinical trial data. This state of play as regards access to clinical trial data prior to the introduction of the CTR was thus complex and relatively ambiguous, further complemented by the emerging case law of the Court of Justice of the European Union, without significant improvements to the clarity and integrity of the system. The EMA’s Policy 0070 was suspended shortly after the implementation of its “Phase 1” began, pertaining to publication of clinical reports only.^50^ “Phase 2” was set to standardize access to anonymized individual patient data but has not yet been implemented.^51^ As of today, the Policy remains suspended due to the COVID-19 pandemic.^52^ Policy 0070 constituted a commendable step towards transparency: the clinical trial data publication report on the first year of implementation of the Policy revealed that over 3,000 clinical documents corresponding to regulatory procedures for 50 medicines were made publicly available, with CCI redactions in the documents published amounting to only 0.01% of the total pages published.^53^

As Kim^54^ describes the scope of accessible clinical trial data in the European Union, it largely depends on the interpretation of the notion of commercially confidential information, as derived from the exception to the fundamental right of access to documents (Art. 15(3) TFEU and Art. 42 of the EU Charter of Fundamental Rights). Access to clinical trial data has been granted with the exception of CCI in conjunction with the general assumption that “clinical data cannot be considered CCI”^55^ except in limited circumstances. However, there has been no consensus in the judicature on the specifics as to the scope of clinical reports subject to confidential treatment. Kim concludes that with no clear legal test of applying the CCI exception, “the rules of access do not present a systemic solution that could provide legal certainty”.

The pharmaceutical industry’s concerns regarding the potential misuse of disclosed information by competitors in support of third-party medicinal product authorization applications without the consent of the sponsor/MA holder have resurfaced following the introduction of the CTR, particularly in connection with the introduction of requirements for earlier publication of clinical trial data. However, changes introduced by the CTR should not raise such concerns: first, the CTR does not change the requirements or timing of access to CSRs. As established by the CJEU’s case law and in accordance with the EMA’s Policy 0070, CSRs are to be publicly available following a decision (irrespective of whether the decision is positive or negative, or whether the application itself is withdrawn) reached in the marketing authorization procedure, and redactions of CCI made within the document are to be kept to a minimum. Second, the CTIS does not, unless otherwise decided by the sponsor itself (Art. 37(4) CTR), include raw data (IPD) required in the MA procedure. In this respect, Policy 0070 grants much wider public access than the CTR.

Third, CSRs are submitted to the database only after an MA is granted. At this point, the data disclosed therein cannot be used for another marketing authorization application without the consent of the original data holder due to the system of regulatory exclusivities, which establishes periods of exclusive use of data provided to regulatory agencies – regardless of whether these data are in the public domain or not.

In this regard, Art. 39(3) TRIPS^56^ sets a minimum standard, requiring protection for regulatory data that were necessary for obtaining marketing authorization for a medicinal product and remain undisclosed. However, neither Art. 10(a)(iii) of Directive No. 2001/83 nor the corresponding Art. 14 of Regulation No. 726/2004 limit data and market exclusivity only to *undisclosed* results of tests and trials. In other words, the EMA and national authorities should not accept data generated by another entity during the period of data exclusivity, unless the data holder enters into an agreement for the use of such data (“Letter of Access”^57^). Disclosed or undisclosed clinical trials data cannot be used to support a generic, hybrid or full application before the eight-year data exclusivity period expires for reference and paediatric medicines, and before the expiry of 10 years of market exclusivity for orphan medicines.^58^ After the expiry of said exclusivity periods, the disclosed data can be used in the abridged procedure. After 10 years of an active substance being in well-established use, its disclosed data can also form a part of a bibliographic application for another medicinal product on the grounds of Art. 10a of Directive 2001/83.

In addition to that, the research-oriented industry expressed concerns that competitors with access to full data sets on approved drugs could seek to register identical drugs in countries without strong regulatory data protection regimes. The counterargument could be, however, that, in practice, most of those countries do not require a detailed pharmaceutical dossier any way. For example, in the case of India, draft guidance issued in 2011 suggests that if a drug has been approved in jurisdictions such as Australia, Canada, the European Union, Japan, the United Kingdom, and the United States, approval in India is in fact based largely on approval in these other countries.

## The Principle of Disclosure in the CTR

As a continuation of the rules on transparency discussed above, the EU Clinical Trial Regulation entered into force on 16 June 2014 and became applicable on 31 January 2022. One of its key objectives has been to strengthen the transparency of clinical trial data. Simultaneously, the EU legislature attempts to strike a balance between the need to protect public health and the need to foster the innovation capacity of European medical research.^59^ Although, as indicated above, the CTR is not the first EU policy initiative aimed at increasing public access to clinical trials data, it substantially shifts the transparency requirements to earlier phases of clinical trials, establishing transparency needs throughout the whole process of clinical development, whilst previous efforts were focused on access to dossiers of already authorized medicinal products after this process had been completed. Whereas Policy 0070 concerns data submitted to the EMA for marketing authorization through the centralized procedure (after 1 January 2015), the CTR transparency requirements apply to data generated during the clinical trials approved under the new Regulation – from the trial registration phase, through the start-up and maintenance of the trial phase until the reporting phase, regardless of whether the trial was intended to be used for obtaining marketing authorization for the investigational medicinal product. In this regard, the EMA’s Policy 0070 was said to “serve as a useful complementary tool ahead of the implementation of the new Clinical Trials Regulation”.^60^

Additionally, the CTR introduced the Clinical Trials Information System, set up by the European Medicines Agency in collaboration with the Member States and the European Commission. The system went live on 31 January 2022 and is subject to a three-year transition period.

Under the CTR, all data and information relating to clinical trials entered into the CTIS shall be publicly accessible, unless exceptions specified in Art. 81(4) of the Regulation can be applied. Confidentiality can be justified on the following grounds:

- protecting personal data in accordance with Regulation (EU) 2018/1725;
- *protecting commercially confidential information*, in particular by taking into account the status of the marketing authorization for the medicinal product, unless there is an overriding public interest in disclosure;
- protecting confidential communication between Member States in relation to the preparation of the assessment report;
- ensuring effective supervision of the conduct of a clinical trial by Member States.

One of the objectives behind the principle of disclosure in the CTR is to foster innovation in order to develop novel products and research into new and better uses of existing products.^61^ In this regard, the CTIS acts as a knowledge management resource, stimulating and accelerating further research by building on accumulated knowledge and technical ability, thus going a step further than the EMA’s Policy 0070, the goals of which were limited to enabling public scrutiny and application of new knowledge in future research in the interest of public health.^62^

To this extent, the Regulation mandates that all clinical trials should be registered in the CTIS prior to being started (see Recital 67 and Art. 5(1) CTR^63^) and requires a minimum set of data to be made public on each trial, which encompasses: (a) the main characteristics of a clinical trial,^64^ (b) the conclusion on Part I of the assessment report for the authorization of a clinical trial, (c) the decision on the authorization of a clinical trial, (d) the substantial modification of a clinical trial, and (e) the end date of the trial, with reasons for which trials are ended prematurely where applicable, and, 12 months later, the summary of results and a summary in lay language.^65^

These data, pursuant to the Regulation, should not, in general, be considered confidential. In line with the transparency principle of the CTR, it is important to support public confidence in the clinical trial process and the EU regulatory system for a number of reasons: *inter alia*, to improve EU citizens’ willingness to participate in clinical trials, which is an essential part of medical progress, and to provide trial subjects with information on the trials in which they have participated.

The concept of protecting commercially confidential information is applied within the CTIS via two mechanisms: the deferral mechanism, which will be discussed in detail below, and the option to exempt CCI from publication by making redactions. As part of a clinical trial application and during the clinical trial life cycle, the sponsor submits a document version “for publication” – which should not contain CCI and personal data – and a version “not for publication”.^66^ The two mechanisms are intertwined in such a way that the document version “for publication” is the one that is published at the time designated by a potential deferral.^67^

In principle, the application dossier (which, in general, contains information on the quality, safety and efficacy of the investigational drug, or its proposed therapeutic uses) filed by the sponsor at a time when it seeks approval for its clinical trial is not made public until a decision on the clinical trial has been made.^68^ All application dossiers are subject to publication rules: the data and documents of the initial application, as well as any subsequent applications, e.g. substantial modifications, will be available in the public domain (their contents are subject to redactions to the extent that they are deemed justified). Publication of these data and documents occurs at the designated time in line with deferral rules.^69^ However, pursuant to Art. 81(5) CTR, data contained in the application dossier may become publicly accessible earlier if there is “an overriding public interest in disclosure”.

Finally, for clinical trials intended to be used for obtaining marketing authorization in the EU, Art. 37(4) CTR requires that the clinical study report must be submitted to the CTIS within 30 days after the day the MA has been granted, the procedure for granting marketing authorization has been completed, or the applicant has withdrawn the application. Recital 68 further reinforces that, in general, the data included in a clinical study report should not be considered commercially confidential once one of the three above-mentioned conditions has been met.

It needs to be underlined here that the CTR does not revolutionize the rules of disclosure for CSRs. In accordance with the principles developed in the CJEU’s case law and the EMA’s policies, CSRs should be publicly accessible by either a proactive or reactive mechanism after a decision on marketing authorization is reached. Under the CTR, applicants for marketing authorization are required to submit CSRs to the database within 30 days of a decision on the application. It is the CSR that is considered the most relevant document in terms of access to clinical trial data under the CTR: the importance of disclosure of the CSRs stems both from the role they play in regulatory proceedings (as documents forming a basis for the decisions of regulatory agencies, and thus having significant value in terms of forming public trust and awareness, as well as enabling scientific verification) and the role they may fulfil in terms of building the competitive position of other clinical trial sponsors who benefit from the body of knowledge contained therein. Specifically, the report contains all data collected in the study, some of which may allow other sponsors to tailor or adjust their planned trials and perform additional analyses for only a certain subgroup. For instance, detailed data on the control group in the study may indicate the effectiveness/safety of the “standard of care” therapy, whereas detailed data on criteria of inclusion in or exclusion from a trial and information on adverse events or additional drugs used during a trial can streamline and accelerate competitors’ work.

Thus, notwithstanding the discerned need for transparency, the overall goal of fostering pharmaceutical innovation cannot be achieved without recognizing the legitimate economic interests of sponsors, as innovation and development first require substantial investment in research. Certain information relating to the trials are therefore considered to be commercially confidential, for a certain period of time, in order to allow the commercialization of the newly developed medicinal product.

## Rules of Protection for Commercially Confidential Information in the CTR

The CTR introduces a concept of “*commercially confidential information*” (Art. 81(4)(b)). The Regulation does not provide a legal definition of this notion. The EMA implementing guidelines provide that CCI is to be understood as (a) any information contained in the data or documents submitted to the CTIS database, (b) that is not in the public domain or publicly available, and (c) where disclosure may undermine the legitimate economic interest or competitive position of the clinical trial sponsors or marketing authorization applicants/holders.^70^ According to the EMA’s Appendix on disclosure rules, the latter prerequisite refers to the instances where the sponsor intends to seek marketing authorization for the investigational medicinal product^71^ and to situations where information derived from a trial may contribute to the obtaining of future research funds.^72^ Rules regarding the scope of CCI are not based on the nature of the sponsor organization conducting the trial (commercial, non-commercial or academic), but rather on the “nature” of the trial and “status” of the medicinal product being studied, though they do not specify what either of those terms encompasses.^73^ The conceptual scope of these terms is of the highest importance for the questions posed in this paper, as the protection of CCI is one of four justifications for deviation from the principle of transparency of data in the CTIS, both for making redactions in the “for publication” versions of documents submitted to the CTIS and for applying for deferral of the documents.

The EMA published a draft guidance document on how to approach the protection of personal data and commercially confidential information in documents uploaded and published in the CTIS in April 2022.^74^ In terms of the definition used within the document, it refers to the guidance document on CCI referenced above. The document further offers examples of data elements and types of information which should not be considered CCI (Sec. 4.5) and presents a short list of specific pieces of information that may have commercially confidential value (referred to in detail below), however, on the understanding that users should not by default consider such types of information to be CCI.

Until the guidance is officially adopted, speculations on the scope and interpretation of CCI under the CTR may be based on the existing practices in Member States and the CJEU’s jurisprudence. The new draft guidance itself points to related policies and guidance documents (cited in this paper), noting that the same principles of handling CCI in other contexts apply (p. 32).

The CTR mandates a prospective harmonized access to clinical trial data pertaining to authorized drugs across European regulatory agencies (“For the purposes of this Regulation, in general the data included in a clinical study report *should not be considered commercially confidential* once a marketing authorization has been granted, the procedure for granting the marketing authorization has been completed, the application for marketing authorization has been withdrawn”^75^). However, the Regulation does not act in retrospect and access to CSRs for drugs already authorized through decentralized and national routes is still hampered, especially in light of Policy 0070’s suspension.^76^

The CTR takes a more cautious approach to the issue of protecting commercially confidential information than the EMA’s Policy 0070, which states that, in general, “clinical data cannot be considered CCI”^77^ and only in “limited circumstances”^78^ can any information constitute CCI. In assessing these limited circumstances, the EMA takes into account, for instance, the nature of the product concerned, the competitive situation of the therapeutic market in question, the approval status in other jurisdictions, the novelty of the clinical development, and new developments by the same company. In this regard, Policy 0070 allows for redactions of clinical reports, subject to the Agency’s approval upon assessment of the justification provided by the applicant.

The obligatory justification, as explained in the guidance on the implementation of Policy 0070,^79^ requires the following components:

- it has to highlight the innovative features of the information in the context of the common knowledge within the specific scientific area;
- it has to indicate explicitly to which on-going development programme the proposed redaction relates;
- it has to explain how the disclosure of the information concerned would undermine the applicant’s legitimate economic interest.

In practice, what may be accepted as CCI under the EMA’s policies is a matter of debate and requires a case-by-case assessment. Data from 2018 show that, after one year of Policy 0070, justifications in support of proposed CCI redactions were rejected in 76% of all instances, whereby the most frequent reason for rejection was insufficient justification.^80^ In the experience of one of the biggest pharmaceutical companies, items accepted as CCI under the EMA’s policies mostly concerned *manufacturing details and immunological bioassay specifications*.^81^

The newly published EMA’s draft guidance^82^ lists examples of information that may have commercially confidential value for CTIS users – including clinical trial sponsors or marketing authorization applicants/holders – although the list is not to be understood as by default:

- the names of manufacturers or suppliers of the active substance or the excipients, unless disclosure is required as per current pharmaceutical legislation (e.g. for some biological products);
- the excipients quantitative composition of the investigational/authorized product;
- detailed information on the synthesis or manufacture of the active substance;
- detailed descriptions of the manufacturing and control processes for the investigational/authorized final product;
- information related to future development plans for indications other than the one under investigation and not yet disclosed in the public domain;
- new biomarkers or novel methodologies not yet qualified (to the extent that the information is not yet disclosed in the public domain);
- detailed information concerning innovative analytical methods;
- detailed information on the facilities and equipment available at the sponsor and clinical sites.

The EMA’s “Draft Guidance on how to approach the protection of personal data and commercially confidential information in documents uploaded and published in the Clinical Trial Information System (CTIS)”^83^ is the first official document that aims to ensure a common understanding of what may be considered CCI under the new Regulation and, specifically, in reference to documents submitted to the CTIS, and to increase consistency in the CCI identified across various types of information. It recommends that the guidance is read in conjunction with Policy 0070 and Policy 0043, as well as other guidance documents prepared by the EMA, as the same principles apply.

The EMA proposes a three-step approach to identifying CCI.^84^ In step 1, the applicant is asked to identify whether the information is in the public domain. Step 2 consists of determining whether that piece of information is innovative. Finally, according to step 3, despite the fact that the information is not innovative, it may be considered CCI if its disclosure may undermine the economic interest or competitive position of the owner of the information. The determination whether a given piece of information is *innovative* should be performed in collaboration with experienced professionals having relevant expertise in the clinical research area and should take into account whether the release of such information could undermine the economic interests or competitive position of the owner of the information. Pieces of text contained in clinical trial documents considered not to be of a novel nature include approaches built upon logic and common sense in line with the content of publicly available documents (e.g. scientific literature, guidance documents, treatment management guidelines, etc.).

Under the CTR’s disclosure rules, in general, the data included in a clinical study report should not be considered commercially confidential once a marketing authorization procedure is finalized (see Recital 68). Documents that have been identified by the EMA as those that could potentially contain details of a commercially confidential nature for clinical trials conducted before an MA is granted, or for new indications or formulations of a product already on the market, include:

- protocol, as a document containing extensive detailed information on the Investigative Medicinal Product (IMP), its mode of action and plans for its testing;
- subject information sheet, as containing details of the medicinal product, the purpose of the trial and its objective, as well as tests that will be undertaken;
- list of questions from the Member States, responses and assessment reports, which contain a detailed analysis and critique of the protocol, subject information and the purpose, design and supporting data of the trial;
- investigator brochure, as it contains extensive details on the pre-clinical and clinical testing and development of the investigational medicinal product, as well as further lines of investigation for future development;
- Investigational Medicinal Product Dossier (IMPD), especially its Q section which provides extensive details on the manufacturing methods and controls, the chemical or biological characterization of the product, its stability, stage of pharmaceutical development and further plans in that respect.^85^

Due to the complex nature of these documents, the EMA took the view that requiring sponsors to provide their “for publication” and “not for publication” versions, which would imply applying extensive CCI redactions and justifications thereof, would impose a significant burden both on sponsors and the Member States concerned.^86^ Thus, the CTR introduces a deferral mechanism.

## The Deferral Mechanism

The clinical trial information flow in the CTIS begins when the sponsor submits a clinical trial application. Following its evaluation, a decision is reached by each Member State concerned for the application. As a general rule, at this point the data and documents submitted to the CTIS will be made available to the public – unless the sponsor applied for a *deferral* at the time of the initial application.^87^ When granted, a deferral will delay the publication of specific documents and its extent depending on the selected trial category and type of the document itself.

The deferral mechanism for publication of data and documents in relation to conducting a clinical trial is justified by the Regulation on the grounds that investors need to be able to benefit from their engagement in the new system.

As stated above, at the application stage and during the life cycle of the clinical trial, the sponsor submits “for publication” and “not for publication” versions of documents. The document version “for publication” is the one that is published at the time designated by a potential deferral and should not contain any CCI or personal data.^88^ The ability to redact some parts of the “for publication” versions of documents is considered to be justified in “limited circumstances”,^89^ as it is envisaged that most of the elements considered to be CCI at the time of the clinical trial application submission will no longer be considered CCI when the deferral period elapses. The sponsors are thus expected to “apply critical thinking”^90^ when deciding which elements of the documents will be considered CCI at the time of publication. The deferral itself is thus conceived as a sufficient method of protecting the sponsors’ economic interests and competitive position.

The documents subject to deferral include: (a) main characteristics of the trial, (b) notifications, (c) subject information sheet, (d) protocol, (e) IMPD S&E sections^91^ and Investigator Brochure, (f) responses to RFI,^92^ (g) clinical trial results summary for an intermediate data analysis, and (h) clinical trial results summary and lay person summary. The publication date of CSRs cannot be deferred.

In practical terms, this means that information, such as: EU clinical trial number; name and address of researcher or company carrying out the trial; outcome of the clinical trial application and date of decision; start and end dates of the trial; start and end dates of participant recruitment; and background information on the principal investigator, is publicly available as soon as a clinical trial is approved and included in the CTIS. However, further information, notably: name of the trial; identity of the investigational medicine; trial design, therapeutic intent and protocol code; objectives and endpoints; participant inclusion and exclusion criteria; details of treatment arms and trial results, is subject to the deferral rules described in detail below.^93^

Disclosure of clinical trial data during the pre-registration phase has far-reaching consequences for sponsors of clinical trials intended to be used for obtaining marketing authorization for the investigational medicinal product. First, it can raise issues in terms of safeguarding the novelty and non-obviousness of the invention and requires a well-thought-out early patenting strategy.^94^ Second, the structure of phase I and II studies, as well as the development of the clinical protocol itself is valuable know-how of the sponsor, which allows considerable insight into the developed trial design and may reveal directions for the development of new active substances or new applications of known substances, which constitutes information of strategic importance.

The timing of publication of these documents is calculated with regard to key milestones of each trial: (1) decision on the trial, (2) end of the trial, (3) 12 months after the end of the trial, and (4) an additional milestone set up to seven years after the end of the trial for category 1 trials and up to five years after the end of the trial for category 2 trials (see Table 1 below).^95^

**Table 1:** Deferral rules for the publication of clinical trial data. Source: authors’ own elaboration on the basis of information contained in: EMA (2021)

Actor	Type of information	Category 1 Phase I & equivalence studies	Category 2 Phase II & III	Category 3 Phase IV
**Sponsor**	Main characteristics	Publication of final summary of results	No deferral	No deferral
**Sponsor**	Notifications	Publication of final summary of results	No deferral	No deferral
**Sponsor**	Subject information sheet	Up to 7 years after the end of trial in EU/EEA	Up to 5 years after the end of the trial in EU/EEA	No deferral
**Sponsor**	Protocol	Up to 7 years after the end of trial in EU/EEA	Up to 5 years after the end of the trial in EU/EEA	Publication of final summary of results
**Sponsor**	IMPD S&E sections and Investigator Brochure	Up to 7 years after the end of trial in EU/EEA	Up to 5 years after the end of the trial in EU/EEA	Publication of final summary of results
**Sponsor**	Responses to RFI	Up to 7 years after the end of trial in EU/EEA	Up to 5 years after the end of the trial in EU/EEA	Publication of final summary of results
**Sponsor**	Clinical trial results summary for an intermediate data analysis	1. 12 months after interim analysis date 2. Up to 30 months after the end of the trial in the EU/EEA	No deferral	No deferral
**Sponsor**	Clinical trial results summary and lay person summary	1. 12 months after the end of trial date in the EU/EEA 2. Up to 30 months after the end of the trial in the EU/EEA	No deferral	No deferral

The specific periods set within the deferral mechanism are considered to be adequate for the protection of sponsors’ economic interests: five years has been taken as a mid-point in the average development of a new medicine (generally considered to be 10 years), whereas seven years is set for category 1 trials which often start earlier in development and are generally shorter in duration.^96^ The deferral and consequent timing for publication of the clinical trial data depend on the selected trial category. In this regard, the Member State concerned can require sponsors to modify the chosen trial category or the proposed deferral timing.^97^ Maximum delay in public access is therefore set at seven years for category I trials,^98^ five years for category II (phases 2 and 3), and one year for category III (phase 4 and low-intervention).^99^ The stakeholders’ position during the consultations on the deferral mechanism^100^ was that CSRs referring to Phase I often contain information that has to be kept confidential to avoid the perception of such information as prior art, i.e. information known publicly before the filing date of a patent application, and that they often contain information that reveals the development strategy of the sponsor. The need to lengthen the initial 12-month period of possible deferral after the end of Phase I was also pointed out in the context of filing a patent application.

Taking into account the average length of the individual phases of clinical trials and the duration of the entire marketing authorization procedure, it appears that the maximum periods of possible deferrals are calculated in such a way that their completion falls close to the time of the decision on marketing authorization.

The final results of a clinical trial, in the form of a summary of results, which is subject to deferral rules, shall be publicly available either when the agreed timelines for publication are reached (a maximum of 2.5 years after the end of the trial) or when the trial results are used in an application for marketing authorization and a decision regarding that application is made or the application is withdrawn. In the latter case, the availability of the clinical study report will trigger the publication of the deferred data and documents.^101^

Sponsors may request a deferral at the time of submission of an initial application via the CTIS. Following the evaluation of the application, a decision on whether the trial is authorized, authorized with conditions or not authorized is issued by each Member State. After the decision has been issued, the default is to make all data and documents public at the first opportunity, unless the sponsor has applied for deferral.

With regard to the above, however, it must be pointed out that some information is made publicly available regardless of the trial category and even in case of deferral, namely: EU clinical trial number, sponsor name and address, nature of clinical trial (e.g. bioequivalence in 24 healthy volunteers), decision outcome on the trial application and date of decision, date of start of the trial, dates of start and end of recruitment, date of end of the trial in the Member State(s) in the EEA, and globally (including early termination of the trial), principal investigator curriculum vitae, and suitability of the facilities.^102^

Save from the above, the sponsors remain under obligations set out in the EMA Policies 0043 and 0070.^103^ It must be recalled, however, that the implementation of the EMA Policy 0070 currently remains suspended due to the pandemic.

Deferrals forming an exception to the general rule of disclosure do not apply where there is an overriding public interest in disclosure. As the EMA’s guidelines state, the public interest *per se* is multifactorial, but includes access to information that supports the objectives for transparency: supporting public confidence in the clinical trial process and in the EU medicines regulatory system, providing access to information to facilitate participation in trials, and providing the public with information on clinical trials conducted in the EU that relates to medicines available on the market and on the data used to support decisions on marketing authorization, or use in practice.^104^ Under the first year of Policy 0070’s implementation, CCI redactions have been rejected in 57 instances (out of 454 instances where CCI was proposed) due to the public interest in disclosure, although the Agency has only provided statistical data in this regard, without disclosing details of particular cases.^105^ So far, on the basis of the pre-CTR guidelines, the public interest premise has been perceived by the European Ombudsman as clearly superior to any commercial interest (“public health should always trump commercial interest”,^106^ and “the public interest in disclosure will generally defeat any claim of commercial sensitivity”^107^), whereas the General Court emphasized, in contrast, that the disclosure requires a “delicate assessment” of the interests involved.^108^

There is no guidance on what can constitute an overriding public interest under the CTR. The one example provided by the EMA’s guidance document foresees a “very serious safety incident” such as occurred in the clinical trial of TGN1412.^109^ Each case will require a decision made by the Member State concerned by the trial, supported by the EMA and the EU Commission.^110^ Items made available in these exceptional circumstances would occur via manual override in the CTIS.^111^

Therefore, a conclusion may be formulated that the CTR’s principle of transparency is subject to four exceptions, and the exception that concerns CCI is further subject to an imprecise and unclear exception of an overriding public interest in disclosure.

In the context of the COVID-19 pandemic and in the light of the controversies that have related to lacking instruments of compulsory data-sharing within the framework of regulatory exclusivities,^112^ health emergencies, especially on a global scale, should certainly be considered such an overriding public interest. We put forth that this assumption is in line with the EMA’s decision to implement exceptional measures to maximize the transparency of its regulatory activities on treatments and vaccines for COVID-19 by shortening its standard publishing timeframes and publishing information it does not normally publish for other medicines.^113^

The matter at hand has been clarified by the adoption of Regulation 2022/123 on a reinforced role for the European Medicines Agency in crisis preparedness and management for medicinal products and medical devices.^114^ Whereas rules established by the EMA generally allow the publication of trial protocols to be deferred, Art. 17(1a) of Regulation 2022/123 introduces an exception to be applied “for the duration of a public health emergency” – a situation recognized as such by the EU in accordance with Decision No. 2022/2371 on serious cross-border threats to health^115^ according to which the protocols of trials with the potential to address public health emergencies need to be published in the CTIS at the start of each trial. Additional rules for transparency are established in Art. 17(2) for medicinal products of relevance to the public health emergency which have received marketing authorization – in such a case, the Agency publishes, *inter alia*, the clinical trial data submitted to the EMA in support of the application, where possible within two months of the MA. Interestingly, Regulation 2022/123 has equipped the EMA with the power to “anonymize all personal data and *redact commercially confidential information*” contained in those clinical trial data (Art. 17, last sentence), without specifying which guidelines shall be applicable for the determination of what constitutes CCI in such a situation, nor whether the decisions on redactions would be made upon request by the MA holder or of the EMA’s own motion.

## Enforcement of the New CTR Rules on Disclosure and Deferrals

As the decision concerning the deferral of publication of data and documents containing CCI is left up to the NCAs, it remains to be seen how they will interpret both premises enabling deferrals and the criterion of the overriding public interest. The applicable HMA/EMA guidance document provides leeway for NCAs to exempt release of trial data if they find support in national jurisdiction: “Notwithstanding this guidance document it should be noted that National Competent Authorities/EMA have to follow their national/European legislation in terms of access to documents and on the protection of personal data (based on the EU Directive 95/46/EC)”.^116^

The NCAs will be fully obliged to adhere to European transparency standards from 31 January 2025 onward, when all clinical trials must be regulated under the so-called EU-CTR.^117^ However, even taking into account the EMA’s future guidance documents, it seems that national authorities will have the final say in the decision on deferral of publication. The interpretation of policies and guidelines may thus differ in particular countries, which may in turn leave room for diverging decisions on publication of certain clinical trial data or its deferral.

Essentially, the EMA’s rules on deferral allow clinical trial sponsors to defer certain trial information for up to 10 years after the end of a trial by establishing a complex classification system without providing clear criteria on the prerequisites for derogation from the transparency principle. Importantly, it seems as though the EMA’s interpretation of the role and balance between redactions and deferrals has undergone a significant change compared to the 2015 guidelines. A Q&A document published by the Agency in December 2022 shifts the emphasis from the use of deferrals back to the functionality of redactions. At present, sponsors are “encouraged to submit trial documents with relevant redactions, instead of requesting long deferrals for their publication”, out of concern that “extensive deferrals could significantly reduce the utility of clinical trial data in CTIS”.^118^ The Agency stresses that such redactions must be done in a sufficiently restricted way, so that the redacted documents “remain meaningful to the public, including potential trial participants and health care professionals”.^119^ The practice of the NCAs developed to date consists of granting deferrals in the durations suggested by the sponsors solely based on the trial category that the specific trial falls under (see Table 1).^120^ This means that the 2015 guidelines established by the EMA’s Appendix on disclosure rules, which foresaw a range of deferrals to be granted (given the wording “up to x years” that was used), are not being applied in full. In these circumstances, it seems necessary to either develop specific guidelines for modulating the duration of deferrals or to modify the general rules so that the practice reflects established principles as, in light of the above, it could be stated that the deferral system essentially forms a rule of confidentiality rather than a derogation from the rule of transparency.

Finally, another issue arises in terms of effective access to clinical trial data, namely the uncertainties as to the enforcement of the requirement to post the information in the CTIS. As the enforcement rests with individual Member States, it is up to them to adopt/publish plans to actively monitor companies, impose fines or otherwise enforce this rule (Art. 94 CTR). Whereas it has been reported that some countries plan to impose fines and sanctions for violations of transparency laws,^121^ others – as in the case of Poland – have limited their efforts to only establishing criminal provisions applicable in case of non-compliance with the provisions laid down in the CTR on subject safety.^122^ The latter solution is in conflict with the requirements of Art. 94(2)(a) CTR, which mandates that rules on penalties applicable to infringements of the Regulation address, *inter alia*, “non-compliance with the provisions laid down in this Regulation on submission of information intended to be made publicly available to the [CTIS] database”.

## Conclusions – the True Impact of CTIS on Transparency of Clinical Trial Data

The CTR significantly transformed the EU regulatory framework for access to clinical trial data, previously governed by the EMA’s regulatory measures – Policies 0043 and 0070, which gave effect to the general rule of access to documents as provided for in Regulation (EC) 1049/2001.

Concurrently, the new legislation should not be understood as a direct revision of existing measures, as the scope of its application is different from the EMA’s policies: whereas the policies covered centrally authorized products only, the CTR’s scope includes investigational medicinal products regardless of whether they have marketing authorization – this means that any rules on transparency, disclosure, deferrals and redactions in terms of clinical trial data are now to be uniformly applied by Member States as the process of assessment and supervision of clinical trials is harmonized by the CTR throughout the EU.

Other significant differences include the clinical studies covered (clinical studies submitted to the Agency in the context of marketing authorization procedures versus only clinical trials conducted in the EU and paediatric trials conducted outside the EU that are part of paediatric investigation plans) and documents subject to disclosure (*all* clinical-trial-related information generated during the life cycle of a clinical trial is to be made public in the CTIS, whereas access under the EMA’s policies was restricted to clinical overview, clinical summaries and CSRs, as well as the anonymization report). Nevertheless, its implementation may well improve currently established practices. For example, a newly published study by Paludan-Müller et al. shows that the median time for publication on the basis of Policy 0070 was 511 days and only 4% of the clinical trial data packages were published within the planned timeline.^123^ In this regard, the CTR’s new rules on automatic publication of CSRs in the CTIS will certainly improve the EMA’s performance.

The CTR significantly changed the rules on disclosing clinical trials when it comes to disclosing all clinical trials, not only the successful ones. Before it came into force, third parties could have access to clinical trial data only following conclusion of the regulatory decision-making process within the framework of centralized marketing authorization procedures.^124^ It left a broad range of conducted clinical trials beyond public access, which had obvious negative consequences for the public health interest with regard to public scrutiny and the possibility of conducting secondary analysis of data. Data on failed trials must now be made public in accordance with the general rules on disclosure, which in this context, however, are not without their drawbacks: although intended to significantly streamline and improve access, they can effectively be overridden by the allowed exceptions in the form of the deferral mechanism and redaction. An adjustment to the scope of these exceptions for unsuccessful trials should be considered to better reach the goal of mitigating their potential duplication.

The CTR’s impact also resolves an important constraint that was said to affect sponsors, investigators, ethics committees and journal editors of phase I studies under the Clinical Trials Directive.^125^ Specifically, unless the study was registered elsewhere, no phase I study enrolled in EudraCT (the platform used to upload applications and trial results under the former Clinical Trials Directive) was registered publicly. The CTIS introduces public transparency for phase I studies in the EU, aligning the EU clinical trial regulations with the clinical trial registration policy of the International Committee of Medical Journal Editors and the World Medical Association’s Declaration of Helsinki.^126^

The rules envisaged by the new legislation need to be assessed in two important contexts: the meaning of the system for the public health interest and its relevance for the balanced framework of protection of economic interests and the competitive position of various stakeholders in the pharmaceutical market. As far as the latter are concerned, the criteria for protection of data as commercially confidential information and thus subject to redactions are fairly broad, encompassing undisclosed information that is either innovative or otherwise economically critical for a sponsor or an MA applicant. Assuming that sponsors are granted maximum available durations of deferrals (*inter alia*, the decisions of Member States being made solely based on trial category), meaningful information in the form of clinical trial results summaries will not appear in the CTIS until around the time of a decision in the MA proceedings, which is when the prolific data contained in CSRs are made public by default, as was the case under Policy 0070 (due to be resumed any moment now and encompassing an even wider scope of published data, including IPD). Nevertheless, these documents will not disclose CCI, which remains redacted indefinitely.

When assessing the newly established rules on publication of documents in the CTIS from the point of view of transparency of clinical trial data for the sake of the public health interest, it is vital to stress that requiring sponsors to make decisions on deferrals and redactions prospectively at the time of submission of an initial application, without establishing a corrective mechanism within which these decisions could be altered depending on various circumstances, contradicts the assumed flexibility of the introduced mechanisms for CCI protection. Meanwhile, the flexibility of deferrals and the possibility of making an adequate decision on their duration were intended to improve the transparency of documents generated in the life cycle of clinical trials and reduce the need for extensive redactions. This issue is further exacerbated by a current lack of specific guidelines for Member States as the parties responsible for accepting the proposed redactions and deferral times. Thus, as it currently stands, the deferral system essentially forms a rule of confidentiality rather than a derogation from the rule of transparency.

The transparency rules adopted in the EU-CTR system constitute an important part of the regulatory data protection system and modify the existing framework of regulatory data confidentiality. Despite the expressed concerns of the research-based pharmaceutical sector, the modifications it introduced have not changed the balance in the market between its various stakeholders, in particular innovators and generics. First, the system of deferrals and redactions allows CCI to be protected very effectively and recognizes the need to protect the legitimate economic interest and the competitive position of sponsors/MA applicants. Second, the CTIS does not include data necessary for a marketing authorization application and, with regard to data published after a decision on an MA is made, they are protected by existing periods of data and market exclusivity. Concurrently, earlier disclosure of basic clinical trial information requires vigilance and the use of well-thought-out early patenting strategies. Patent protection will be important in situations of prolonged lead times for the subsequent phases of a clinical trial that result in the disclosure of, for example, a phase I trial protocol well before the MA is obtained. This may be caused due to substantive issues (e.g. the occurrence of strong side effects and the need to halt the trial) or organizational issues (e.g. in the case of the need to find an investor, license or sell the rights to a substance before the start of a phase II trial).

The available anecdotal and rhetorical observations available in the literature point to the conclusion that the CSRs and other regulatory documents may, even including redactions and anonymization, facilitate successful secondary data analysis in academic research.^127^ Taking the use of AI for the secondary analysis of data as an example, one must consider its specific data needs. Clinical study reports – even in searchable formats – are a suboptimal source of data for AI models, creating barriers for data analysis, as other tools must be used to first extract *actionable* data. It could be theorized that the choice of a format for information that is less suitable for most modern, advanced data analysis is connected to the prolonged work on the CTR, which took effect eight years after its text was first published, making it somewhat outdated and unsuited for today’s world of big data. The unfortunate suspension of Policy 0070’s implementation resulted in a lack of empirical evidence regarding the usefulness of individual participant data (IPD) derived from the documents published under the Policy for a more robust secondary analysis. As IPD are essentially complete raw data recorded for each participant in a study, it would be the most suitable source of data for AI, if consistent rules of anonymization are put in place so as not to limit usability. It is the “raw” trial data – individual patient data – that best support confirmatory and exploratory data analyses.^128^

In this regard, the EMA has recently launched a pilot project on the usefulness of conducting analyses of raw data from clinical trials by regulatory authorities in order to improve the evaluation of MA applications and post-authorization applications.^129^ If, as a result of the pilot project, the submission of raw data does become required in the context of marketing authorization, their disclosure as part of the regulatory dossier may remedy the current lack of access to actionable data. The cautiously announced return to Policy 0070 implementation may also provide for a better source of raw data for secondary data analysis than the CTIS. Whereas the CTR only foresees the establishment of guidelines for formatting and sharing of raw data in case the sponsor itself decides to share it voluntarily (Art. 37(4) CTR), Policy 0070’s scope presupposes the release of IPD submitted under the centralized MA procedure in a wide range of instances.^130^ At the same time, wider access to IPD will reinstate the discussion on the protection of the privacy of individuals involved in the trials.^131^

Although the CTR is directly applicable in all EU Member States,^132^ national legislation is required to address certain aspects of the Regulation. As of today, the Polish Act on Clinical Trials of Medicinal Products for Human Use is still being considered by the Senate,^133^ whereas other Member States have already completed the transposition of the CTR into national law.^134^ Ultimately, the value of the information made public in the CTIS for AI cannot be fully assessed at this time, as the actual scope of data available for secondary analysis remains unclear, largely dependent on Member States’ decisions on redactions, deferrals and their interpretation of the notion of an “overriding public interest”. In view of the above, a comprehensive assessment of changes introduced by the Clinical Trials Regulation must be revisited following its complete implementation in national legislation and the CTIS’s functionality having been tested in practice.

Expanding the current state of knowledge on how the system works in practice will also enable a comprehensive analysis of the relationship between the scope of protection of undisclosed know-how, protected as trade secrets,^135^ and the scope of protection of commercially confidential information within the presented legal framework. Perhaps the most meaningful interface between the systems relates to the disclosure of specific data within the CTIS, which, be it obligatory, voluntarily or accidental, deems the data ineligible for trade secret protection. This and many other issues require further careful consideration.

## Footnotes

1 *See*, *e.g*., U.S. Food and Drug Administration (1997), which led to the inception of ClinicalTrials.Gov in 2000.

2 The WHO’s resolution of 2005 similarly led to the establishment of an international trials registry platform – International Clinical Trials Registry Platform (ICTRP), WHO Statement on Public Disclosure of Clinical Trial Results, http://www.who.int/ictrp/results/reporting/en/ (last accessed 13 February 2023).

3 *See*, *e.g*., Chan et al. (2014), Doshi et al. (2012), Terry and Terry (2011), Eichler et al. (2012).

4 Weissler et al. (2021).

5 Regulation (EU) No. 536/2014 of the European Parliament and of the Council of 16 April 2014 on clinical trials on medicinal products for human use, and repealing Directive 2001/20/EC, OJ L 158, 27 May 2014, pp. 1–76.

6 Recital 25 of the CTR.

7 Recital 67 of the CTR.

8 These concerns have prevailed since the first comments on the “Draft proposal for an addendum, on transparency, to the ‘Functional specifications for the EU portal and EU database to be audited’ (EMA/42176/2014)” and the “Draft Appendices to Draft proposal for an addendum, on transparency, to the ‘Functional specifications for the EU portal and EU database to be audited’ (EMA/641479/2014)” were submitted by industry members. *See* EFPIA, EBE, VE (2015). *See also* recent media coverage on a number of questions on transparency aspects raised by sponsors during a survey undertaken last year (*e.g*. Sharma (2023)).

9 *See*, *inter alia*, EMA (2010) and EMA (2019), as well as HMA/EMA Working Group on Transparency (2012) (hereinafter: “Guidance document on CCI”).

10 *See*, *e.g*., Kim (2021), p. 24.

11 Regulation (EU) No. 45/2001 replaced by Regulation (EU) 2018/1725 of the European Parliament and of the Council of 23 October 2018 on the protection of natural persons with regard to the processing of personal data by the Union institutions, bodies, offices and agencies and on the free movement of such data, and repealing Regulation (EC) No. 45/2001; and Decision No. 1247/2002/EC, OJ L 295, 21 November 2018, pp. 39–98.

12 Nolan et al. (2021), p. 3.

13 El Emam and Abdallah (2015).

14 Lemmens (2014).

15 Kim (2021), p. 14. The author also stresses that drug innovation is generally considered to be a highly risky and costly enterprise.

16 Directive 2001/20/EC of the European Parliament and of the Council of 4 April 2001 on the approximation of the laws, regulations and administrative provisions of the Member States relating to the implementation of good clinical practice in the conduct of clinical trials on medicinal products for human use, OJ L 121, 1 May 2001, pp. 34–44.

17 Art. 9(1) and (2) of the CTD and Art. 5 of the CTR.

18 By 31 January 2025, all ongoing trials approved under the Directive will be governed by the CTR and have to be transitioned to CTIS. EMA, Regulatory harmonisation of clinical trials in the EU: Clinical Trials Regulation to enter into application and new Clinical Trials Information System to be launched, 25 February 2022, https://www.ema.europa.eu/en/news/regulatory-harmonisation-clinical-trials-eu-clinical-trials-regulation-enter-application-new (last accessed 13 February 2023).

19 *See also* Kim (2021), pp. 16–19, discussing levels of transparency in clinical trials.

20 Charter of Fundamental Rights of the European Union, OJ C 326, 26 October 2012, pp. 391–407.

21 Art. 15(3): “Any citizen of the Union, and any natural or legal person residing or having its registered office in a Member State, shall have a right of access to documents of the Union’s institutions, bodies, offices and agencies, whatever their medium, subject to the principles and the conditions to be defined in accordance with this paragraph”. Consolidated version of the Treaty on the Functioning of the European Union, OJ C 326, 26 October 2012, pp. 47–390.

22 Art. 2: “Any citizen of the Union, and any natural or legal person residing or having its registered office in a Member State, has a right of access to documents of the institutions, subject to the principles, conditions and limits defined in this Regulation”. Regulation (EC) No. 1049/2001 of the European Parliament and of the Council of 30 May 2001 regarding public access to European Parliament, Council and Commission documents, OJ L 145, 31 May 2001, pp. 43–48.

23 *See* Art. 41 of Regulation (EC) No. 1901/2006 of the European Parliament and of the Council of 12 December 2006 on medicinal products for paediatric use and amending Regulation (EEC) No. 1768/92, Directive 2001/20/EC, Directive 2001/83/EC and Regulation (EC) No. 726/2004, OJ L 378, 27 December 2006, pp. 1–19.

24 *See* Art. 80 of the Regulation, which stipulates that information related to the authorization or supervision of medicinal products shall be made available to the public in order to ensure an appropriate level of transparency. Regulation (EC) No. 726/2004 of the European Parliament and of the Council of 31 March 2004 laying down Community procedures for the authorisation and supervision of medicinal products for human and veterinary use and establishing a European Medicines Agency, OJ L 136, 30 April 2004, pp. 1–33.

25 EMA (2010).

26 EMA (2019).

27 This document is a consensus document agreed by the whole network of National Competent Authorities and as such is intended to be applicable to information requests on medicinal products authorized under the national, mutual recognition, decentralized and centralized procedures. HMA/EMA Working Group on Transparency (2012).

28 *See* the PHUSE (2020) analysis showing that 190 of 253 countries have databases or a registry for the registration and publication of clinical studies.

29 These authorities are later referred to as “National Competent Authorities” or NCAs. *See also* Sect. 3 of this paper for more details on access to regulatory dossiers in individual Member States.

30 *See*, *e.g*., judgment of the Polish Supreme Administrative Court of 18 December 2020 (I OSK 2377/19) in which it was ruled that the refusal of access to documents submitted in the procedure for marketing authorization of a medicinal product by the Polish NCA should be considered as infringing on the principles of loyal cooperation both with regard to the European Union and with regard to other Member States, as the Guidance document on CCI was authored both by the EMA and the Heads of Medicines Agencies of individual Member States.

31 Pursuant to Art. 80 of Regulation No. 726/2004, the EMA adopts rules to ensure the availability to the public of regulatory, scientific or technical information concerning the authorization or supervision of medicinal products which is not of a confidential nature.

32 Gøtzsche (2011) recalls having to wait three years and to file a complaint to the European Ombudsman before he was able to access trial protocols and clinical study reports for two anti-obesity drugs at the EMA.

33 Clinical data released under Policy 0070 normally include: (a) the clinical overview, providing a critical analysis of the clinical data in the submission package, including the conclusions and implications of the clinical data; (b) the clinical summary, which provides a detailed factual summarization of all the clinical information submitted; (c) the study reports on the individual clinical studies; (d) three appendices to the clinical study reports, namely the study protocol, the sample case report form used to record information on an individual patient, and documentation of the statistical methods used to analyze the data.

34 Note that the changes introduced by Policies 0043 and 0070 apply only to clinical data held by the Agency.

35 *See* Guidance document on CCI, pp. 47–54.

36 *See*, *e.g*., Orders of the Vice-President of the Court of 28 November 2013 in *EMA v. AbbVie*, Case C-389/13 P (R) (ECLI:EU:C:2013:794); and *EMA v. InterMune*, Case C-390/13 P (R) (ECLI:EU:C:2013:795).

37 ICH Topic E3 Structure and Content of Clinical Study Reports, July 1996, European Medicines Agency, https://www.ema.europa.eu/en/documents/scientific-guideline/ich-e-3-structure-contentclinical-study-reports-step-5_en.pdf (last accessed 13 February 2023).

38 Doshi and Jefferson (2013). For detailed information on the structure and content of clinical study reports, *see* EMA (1996).

39 *See* Committee on Strategies for Responsible Sharing of Clinical Trial Data, Board on Health Sciences Policy, Institute of Medicine (2015) and submissions to the Committee from the trade organization Pharmaceutical Research and Manufacturers of America (PhRMA) and AbbVie cited therein. Pharmaceutical companies specifically pointed to jurisdictions such as Australia, Brazil and China; however, the merit of their arguments, as stated by the Committee, ultimately largely depend on whether these jurisdictions actually require detailed submissions – for example, approval of medicines in India is in fact largely based on approval by the FDA and the EMA (CDSCO 2011).

40 Rabesandratana (2013); *see also* Ferran and Nevitt (2019), p. 3, for a table with a breakdown of Policy 0043 document requests per affiliation.

41 EMA (2018a), p. 14.

42 *See supra* note 36 and the CJEU judgments referred to therein.

43 EMA, Clinical data website, https://clinicaldata.ema.europa.eu/web/cdp/home (last accessed 13 February 2023).

44 EMA (2019), p. 6.

45 El Emam and Boardman (2018).

46 In 2018, the European General Court ruled in favour of the EMA’s principle of transparency, enshrined in Policy 0043 and Policy 0070, in three separate cases: T-235/13 (*Pari Pharma v. EMA*), T-718/15 (*PTC Therapeutics International v. EMA*) and T-729/15 (*MSD Animal Health Innovation and Intervet International v. EMA*). The CJEU ruled that the marketing applications should not be considered commercially confidential in their entirety, and that release of certain parts (such as the Clinical Study Report) does not violate the companies’ commercial interests. PCT Therapeutics and MSC challenged the EMA’s transparency rules again in 2019; however, the ECJ ruled in favor of the transparency approach again in 2020. *See* Court of Justice of the European Union (2020).

47 Boesen (2021).

48 *See* Guidance document on CCI.

49 Boesen (2021), for Poland, *see* note 31.

50 The EMA begun proactively publishing clinical data submitted in the MA procedure in October 2016 and suspended the policy on 5 December 2018.

51 EMA (2018b), p. 6.

52 *See* the EMA’s website, tab “Support for industry on clinical data publication”, https://www.ema.europa.eu/en/human-regulatory/marketing-authorisation/clinical-data-publication/support-industry-clinical-data-publication (last accessed 13 February 2023). The Agency has nevertheless implemented exceptional measures to maximize the transparency of its regulatory activities on treatments and vaccines for COVID-19 that are approved or are under evaluation, by shortening its standard publishing timeframes and publishing information it does not normally publish for other medicines (EMA’s website, tab “Transparency: exceptional measures for COVID-19 medicines”, https://www.ema.europa.eu/en/human-regulatory/overview/public-health-threats/coronavirus-disease-covid-19/treatments-vaccines/transparency-exceptional-measures-covid-19-medicines (last accessed 13 February 2023). According to the public summary of a December 2022 EMA Management Board meeting, the Policy is expected to be revived in 2023 (EMA, EMA Management Board: highlights of December 2022 meeting, 16 December 2022, https://www.ema.europa.eu/en/news/ema-management-board-highlights-december-2022-meeting (last accessed 13 February 2023).

53 EMA (2018a), p. 2.

54 Kim (2017).

55 *See* EMA (2019).

56 TRIPS: Agreement on Trade-Related Aspects of Intellectual Property Rights (1994) Marrakesh Agreement Establishing the World Trade Organization, Annex 1C, 1869 U.N.T.S. 299, 33 I.L.M. 1197.

57 *See*, in relation to data sharing practices in the agrochemical industry, Lenarczyk and Zemła-Pacud (2023), forthcoming.

58 Art. 10(a)(iii) of Directive No. 2001/83; Art. 14 of Regulation No. 726/2004.

59 Recital 67 of the CTR.

60 *See* EMA (2014b), pp. 6–7.

61 EMA (2015a), p. 3.

62 EMA (2019), pp. 3–4.

63 “In order to obtain an authorization, the sponsor shall submit an application dossier to the intended Member States concerned through the portal referred to in Article 80”.

64 Comprising design, scientific and, where applicable, therapeutic intent, title, identification of the investigational medicinal products (IMPs), treatment arms, treatment population and number of subjects, inclusion and exclusion criteria and main objectives and endpoints. *See ibid*. Note that, according to the EMA’s Appendix on disclosure rules, the publication of the main characteristics of category 1 clinical trials (*see* note 107 for details on which trials fall under this category) may be deferred until the first summary results are made public. EMA (2015a), p. 15.

65 *See* Recital 68 CTR. This requirement has later been specified in the EMA’s Appendix on disclosure rules – a document which sets out rules and criteria for the application of exceptions to the public availability of CTIS established in the CTR. In accordance with the Appendix, the information that will be made public for all clinical trials registered in the system includes: (a) the main characteristics of the trial comprising design, scientific and, where applicable, therapeutic intent, title, identification of the investigational medicinal products (IMPs), treatment arms, treatment population and number of subjects, inclusion and exclusion criteria and main objectives and endpoints; (b) conclusion of the assessment and decision on the trial; (c) information updated during the trial to indicate the start and end dates of recruitment; (d) substantial modifications to the trial; (e) the end date of the trial, with reasons for which trials are ended prematurely where applicable, and, 12 months later, the summary of results and a summary in lay language; (d) clinical study reports for clinical trials on medicines for which a marketing authorization has been granted, the procedure completed or the marketing authorization application withdrawn (EMA (2015a), p. 3).

66 EMA (2022b), p. 43.

67 *Ibid*.

68 *See* Art. 81(5) CTR. This applies to any decision outcome, whether it be an authorization, an authorization with conditions or a refusal.

69 EMA (2015a), p. 6.

70 Working definition of CCI as provided in the Guidance document on CCI (HMA/EMA Working Group on Transparency (2012)), adopted in the EMA’s Appendix on disclosure rules (EMA (2015a), p. 11).

71 The first instance is explicitly mentioned in Regulation No. 536/2014. Art. 81(4)(b) CTR states that the assessment of the justification for protecting CCI must be conducted: “In particular through taking into account the status of the marketing authorisation for the medicinal product”. In accordance with Art. 82 CTR, the Agency is responsible for drawing up the functional specifications for the EU portal and the EU database (published by the EMA in 2015 following public consultation, EMA (2015a)) which includes a document providing further detail on the transparency requirements established by the CTR, referred to in this paper as the “Appendix on disclosure rules”.

72 EMA (2015a), p. 11.

73 EMA (2015b), p. 4.

74 EMA (2022a).

75 Recital 68 CTR.

76 Guidance document accompanying the policy stated that “as a general rule all clinical reports submitted as part of a regulatory application will be subject to publication”. EMA (2018b), p. 6. *See also* Berlin Institute of Health (2021).

77 EMA (2019), p. 4.

78 *Ibid*.

79 EMA (2018b), p. 59.

80 *See* Eibert (2018), p. 35, citing Henry-Eude.

81 The term “immunological bioassay specifications” refers to the results of an analytical method used to determine the relative strength of a substance. Specifications are critical quality standards that are proposed by the manufacturer and approved by regulatory agencies as conditions of approval. *Ibid*., p. 36.

82 EMA (2022a), p. 34.

83 EMA (2022a).

84 EMA (2022a), p. 35.

85 EMA (2015b), pp. 15–17.

86 EMA (2015b), p. 14.

87 EMA (2022a), p. 15.

88 EMA (2022b), p. 31.

89 EMA (2022a), p. 32.

90 *Ibid*.

91 Investigational Medicinal Product Dossier (IMPD) sections S and E provide extensive detail on the manufacturing methods and controls, the chemical or biological characterization of the product, its stability, stage of pharmaceutical development and further plans in that respect. *See* EMA (2015a), p. 28.

92 During the evaluation of a clinical trial application, the Member State concerned can raise a request for information (RFI) to be addressed by the sponsor of the trial.

93 EU Clinical Trials Website, about searching for clinical trials and reports, https://euclinicaltrials.eu/search-clinical-trials-reports/?lang=en (last accessed 13 February 2023).

94 Where it is possible, such a strategy can involve and may benefit from a provisional patent application which allows for drafting claims better corresponding to the newest findings of phase I clinical trials.

95 Some exceptions apply, *inter alia* for category 1 trials that are conducted in paediatric populations or are included as part of a paediatric investigational plan; it is not possible to defer the publication of: main characteristics of the trial, notifications, intermediate data analysis, summary of results and the layperson summary. EMA (2022a), p. 22.

96 EMA (2015a), p. 14.

97 *See* EMA (2022a), p. 20.

98 Category 1 trials are: (a) phase I clinical trials in healthy volunteers or patients, carried out to test whether a treatment is safe for people to take, and to study pharmacokinetics and pharmacodynamics (where possible); (b) phase 0 trials – trials in healthy volunteers or patients, without therapeutic or prophylactic intent, to explore pharmacokinetics or pharmacodynamics; (c) bioequivalence and bioavailability trials of innovative products, new generic products and biosimilar products; (d) similarity trials for biosimilar products; (e) equivalence trials for combination products or topical products where a pharmacodynamic or efficacy endpoint is used to determine equivalence, and where pharmacokinetic and or pharmacodynamic studies are not possible.

99 For a detailed overview of the timing of publication of data and documents from the clinical trial database in relation to the category of the trial, *see* EMA (2015a), pp. 15–19.

100 EMA (2015c), p. 18.

101 In accordance with Art. 27(4) of the Regulation, CSRs are to be submitted to CTIS within 30 days after the day the marketing authorization has been granted, the procedure for granting the marketing authorization has been completed, or the applicant for marketing authorization has withdrawn the application. *See* EMA (2022a), p. 23.

102 EMA (2015a), p. 20. Whereas the new EMA’s draft guidance on how to approach CCI (EMA (2022a, b)) pertains to the protection of personal data and CCI, the EMA’s Appendix on disclosure rules of 2015 is the source of more technical information about the functionality of the CTIS database. They are to be read in conjunction.

103 The EMA has specified that the guidelines relating to CCI under the CTR need to be read in conjunction with the Agency’s previous policies and guidance documents, including Policy 0070 and Policy 0043. EMA (2022a), pp. 32–33.

104 *Ibid*., pp. 4–5.

105 EMA (2018a), p. 7.

106 European Ombudsman (2016), paras. 73–74.

107 *Ibid*, summary.

108 *AbbVie v. EMA*, *supra* note 36, para. 69; *Pari Pharma v. EMA*, *supra* note 46, para. 58.

109 TGN1412 was withdrawn from development after inducing severe inflammatory reactions as well as chronic organ failure in the first-in-human study in March 2006. *See* Goldacre (2012), pp. 8–10, 104–105.

110 EMA (2015a), p. 12.

111 EMA (2015a), p. 8.

112 *See*, *e.g*., Lenarczyk and Zemła-Pacud (2020), pp. 154–155.

113 *See supra* note 33.

114 Regulation (EU) 2022/123 of the European Parliament and of the Council of 25 January 2022 on a reinforced role for the European Medicines Agency in crisis preparedness and management for medicinal products and medical devices, PE/76/2021/REV/1, OJ L 20, 31 January 2022, pp. 1–37.

115 Regulation (EU) 2022/2371 of the European Parliament and of the Council of 23 November 2022 on serious cross-border threats to health and repealing Directive No. 1082/2013/EU, PE/40/2022/REV/1, OJ L 314, 6 December 2022, pp. 26–63.

116 Guidance document on CCI.

117 From 31 January 2022 to 31 January 2023, sponsors could submit clinical trials under the legal framework of EU-CTD or EU-CTR. Starting 31 January 2023, all clinical trial applications are subject to EU-CTR. However, trials approved under EU-CTD before 31 January 2023 can continue to be regulated under EU-CTD until 31 January 2025.

118 EMA (2022c), pp. 59–60.

119 *Ibid*., p. 60.

120 During a CTIS event organized by the EMA, it was stated by the Agency representative that decisions on deferrals are so far linked to category selection and no other bases of rejection of deferral requests have been identified. EMA, “Clinical Trials Information System (CTIS): Readiness for mandatory use of the Clinical Trials Regulation from 31 January 2023” event, 20 January 2023. Recording available at: https://www.ema.europa.eu/en/events/clinical-trials-information-system-ctis-readiness-mandatory-use-clinical-trials-regulation-31 (last accessed 13 February 2023).

121 *See* blog posts on actions taken in Denmark and Belgium: TranspariMED blog, Belgium, Regulator to impose fines for unreported clinical trials, 21 January 2022, https://www.transparimed.org/single-post/belgium-regulator-to-impose-fines-for-unreported-clinical-trials; Denmark to introduce sanctions for non-reporting of clinical trial results, 20 March 2020, https://www.transparimed.org/single-post/2020/03/10/denmark-eudract-clinical-trial-regulation (both last accessed 13 February 2023).

122 *See* wording of Art. 77 of the draft Act of 13 January 2023 on clinical trials on medicinal products for human use, currently under deliberation in the Senate, https://orka.sejm.gov.pl/proc9.nsf/ustawy/2843_u.htm (last accessed 13 February 2023).

123 Paludan-Müller et al. (2022).

124 Clinical reports contained in withdrawn applications are also, in principle, published under Policy 0070, subject to an exceptional situation where there is a confirmed re-submission date or re-submission of the application has already taken place. *See* EMA (2018b), pp. 6–7.

125 De Mey (2022).

126 *See* provisions of the clinical trial registration policy of the International Committee of Medical Journal Editors (ICMJE) (ICMJE Recommendations – Clinical Trials – Registration, available at: http://www.icmje.org/recommendations/browse/publishing-and-editorial-issues/clinical-trial-registration.html); and Secs. 35–36 of the World Medical Association’s Declaration of Helsinki – Ethical Principles for Medical Research Involving Human Subjects, 9 July 2018, available at: https://www.wma.net/policies-post/wma-declaration-of-helsinki-ethical-principles-for-medical-research-involving-human-subjects/.

127 Ferran and Nevitt (2019).

128 Both terms relate to the secondary data analysis, conducted either for the purpose of validating the conclusion of the primary analysis done in accordance with the hypothesis and research questions defined in trial protocol prospectively (confirmatory data analysis) or for the purpose of exploring research questions posed outside this scope entirely (exploratory data analysis). *See* Kim (2021), pp. 48, 50.

129 EMA launches pilot project on analysis of raw data from clinical trials, 12 July 2022, https://www.ema.europa.eu/en/news/ema-launches-pilot-project-analysis-raw-data-clinical-trials (last accessed 13 February 2023). The pilot project is expected to last for up to two years.

130 EMA (2019), p. 2. These instances include IPD submitted: (a) as part of an MA application; (b) or as part of a post-authorization procedure for an existing centrally authorized medicinal product; (c) or as part of a procedure under Art. 58 of Regulation (EC) No. 726/2004; (d) or submitted by a third party in the context of an MA application or a post-authorization procedure for an existing centrally authorized medicinal product; (e) or requested by the Agency or submitted by the applicant/MA holder as additional clinical data in the context of the scientific assessment process for the aforementioned situations.

131 *See*, *e.g*., Minssen (2020); Neethu (2019).

132 Specifically, the CTIS will be the single-entry point for submission of clinical trial applications, authorization and supervision in the EU, and in the European Economic Area (EEA) countries Iceland, Liechtenstein and Norway. Following Brexit, submissions in the UK will be carried out separately via the Medicines and Healthcare products Regulatory Agency.

133 For updates on progress of the draft Act, *see* the Government Legislation Centre’s website: https://legislacja.rcl.gov.pl/projekt/12346302/katalog/12784810 (last accessed 30 May 2022).

134 For example, in Ireland, the European Union (Clinical Trials on Medicinal Products for Human Use) (Principal) Regulations 2022 came into effect on 31 January 2022 (S.I. No. 99/2022), whereas the German Medicinal Products Act (*Arzneimittelgesetz*, AMG) was first amended with regard to the new Regulation on 24 December 2016.

135 Directive (EU) 2016/943 of the European Parliament and of the Council of 8 June 2016 on the protection of undisclosed know-how and business information (trade secrets) against their unlawful acquisition, use and disclosure, OJ L 157, 15 June 2016, pp. 1–18.
